# On the Nature, Cause and Treatment of Cholera

**Published:** 1830-10-01

**Authors:** 


Cholera, its Nature, Cause, and Treatment; with original Views, physiological, pathological, and therapeutical, in relation to Fever, the Action of Poisons, &c. &c. &c. By *Charles Searle*, Surgeon, of the Honourable East India Company's Madras Establishment. Octavo, pp. 256. 1830.

Mr. Searle, we fear, has been rather injudicious. If he had stated his "original views" without encumbering them with a load of quotations from various works, they might have stood some chance of being read, even if they were not acceded to;—but, at this time of day, to make up a book on cholera principally from works recently published on the subject, interweaving a slight tissue of originality, hardly capable of keeping the texture together, was an unadvised undertaking. We grieve to make these observations at the very beginning of our review of the work, but candour compels us to the declaration. We shall endeavour, however, to drag from the great mass of quotations, constituting the majority of the work, as much as we can of those original views which form so conspicuous a feature in the title-page.

The volume commences with a general description of cholera, extracted verbatim from the Report of the Madras Medical Board long since published. This, of course, we shall pass. Next are introduced extracts from Mr. Orton's work, bearing on the progression of the symptoms of cholera. These constitute the first chapter. The second contains the "appearances on dissection," "also extracted from Mr. Orton's work, substantiated by quotations from the best authorities." The third chapter brings us in contact with originality—"the case of the author, with circumstances attending the attack." We shall lay this case before our readers.

"The subject of this case, ætat. 34; 12 years in India; of spare make, and delicate state of health. The 16th of October, 1828. For the past week has felt tolerably well, though for some days past has been subject, on the slightest exposure, to catarrhal attack. The bowels this morning were healthily relieved, and he felt well during the day, dining at 2 p.m. off roast mutton with rice, and drinking therewith three glasses of good sound sherry. At 5 p.m. he took as usual a tepid bath, and subsequently spent the evening in a quiet way at a friend's house, at half a mile's distance: where he made an abstemious supper (usually called dinner) of light soup, with a mouthful off the breast of a snipe, and dried toast; and drank a couple of glasses of good sherry.—It having rained hard an hour before, and the weather being damp and cloudy, he returned home in a palankeen.—On going to bed at 11, he felt well, but a little fatigued, which he attributed to want of rest; his usual hour of retiring being nine o'clock: he exposed himself, as it was his customary practice, to aerial influence, whilst he now rubbed himself well with a coarse towel. On this occasion, the windows being open, as was usually the case, and the weather at the same time damp and raw, he distinctly remembers having felt a passing chill, and a thought at the moment came across the mind, that exposure in this way at all times was hardly prudent: but in getting into bed he soon fell asleep, and continued so till about 1 a.m. when he awoke, suffering from a sense of distention and oppression at the præcordia, which, attributing to flatulency, he endeavoured to relieve by sitting up in bed and rubbing the abdomen; but this affording no relief, he got out of bed, with the intention of taking a little brandy and water—when he immediately felt an urgent propensity to stool, and, as the commode was at hand, almost as instantly passed a copious fluid dejection, without the slightest pain or uneasiness, but with no relief to the abdominal oppression. On leaving the commode, he suddenly felt giddy, which went off on assuming the recumbent posture, but left him sighing, the pulse exceedingly weak, the voice subdued and feeble, the extremities cold, and with a remarkable thrilling sensation, or feeling of nervous tremor in every fibre of the body, but more particularly experienced in the extremities: he now took half a wine-glassful of brandy with double the quantity of hot water; although a little wind was expelled from the stomach, it afforded no relief to the præcordial oppression; he, therefore, swallowed 12 drops of oil of peppermint, in a tea spoonful of sugar; but as no relief was experienced, warm flannels were applied to the belly; these felt hot to the part, but afforded no relief; another copious fluid brown dejection was now passed, which was almost immediately succeeded (I believe from assuming the erect position) by vomiting of a small quantity of mucous fluid with the peppermint: presently afterwards a desire was again felt to stool, but being restrained, the stomach appeared immediately to sympathise, and ejected with some force its contents, consisting of about 6 ounces of ropy mucus, enveloping a small quantity of indigested rice, which tasted very sour. The vomiting occasioned a singing noise in the ears, and sense of great exhaustion, with sighing and oppression of breathing, which was only relieved by the unceasing use of the punkah.* Ten minutes afterwards the following draught was taken.

℞: Ammon. Carbon. gr. x.

Magnesiæ ...... ʒj.

Magnes Sulph... ʒiij.

Aquæ .......... ℥iij. M. ft.

This induced an agreeable warmth in the stomach, but no expulsion of flatus, nor did it afford any relief to the præcordial oppression, which continued unabated, although hot flannels had been constantly applied. Another evacuation was now passed, and soon after a desire was again felt, but being uncomplied with, the stomach became again sympathetically excited, and the draught, which had been taken now about a quarter of an hour, was thrown off, much diluted with a floculent colourless fluid, tasting strong of the ammonia. Vomiting was in this instance attended with a little perspiration, but of very transitory duration; and there was a little relief felt of the præcordial oppression. A few minutes afterwards, a couple of clysters of a solution of salt in warm water were thrown up the bowels, which were succeeded by a copious floculent mucous fluid evacuation: this afforded slight, but sensible relief to the abdominal oppression. Shortly afterwards, on attempting to sit up, faintness came on, followed by vomiting of a mouthful of mucous fluid, singing noise in the ears, and feelings of great exhaustion. The præcordial oppression being still great, the clysters were repeated, and continued at short intervals—as they were ge-

* A fan.—ED.

nerally succeeded by evacuation, which always afforded more or less relief. As the abdomen felt hot to the hand, and there was some thirst now experienced with desire for cold drink—a wine-glassful of cold water was taken, and repeated every half-hour, with great comfort, and no subsequent vomiting. The warm salt water injections at short intervals, with the cold water for drink, and the constant use of the punkah, were continued from this time, about half-past 3, till 6 o'clock—with progressive relief to the præcordial oppression, which still continued considerable, and with general amendment—when an increase of thirst being experienced, with sense of preternatural heat of the extremities, the clysters were discontinued, and a drachm of Cheltenham salts dissolved in 4 ounces of water was taken, and the same dose repeated 2 hours afterwards; by which pretty copious, colourless sero-mucous evacuations were continued to be passed at short intervals, with gradual relief of the præcordial oppression, and comfortable feelings of amendment. At 9, a.m. a cup of tea was taken, but the milk in it soon after becoming sour, occasioned nausea and oppression of stomach, which continued a couple of hours. The heat of skin having progressively increased, at 11 o'clock the following pills were taken, with a claret-glassful of cold water, and the latter was repeated occasionally afterwards, ad libitum.

℞. Hydr. Submur.
Pulv. Antimon. āā gr. ij.
Ext. Coloc. Co... gr. vj. M. ft. Pil. ij.

I must observe, that after 10 o'clock, the evacuations which continued to be passed every half hour or so, became less copious, thicker, and somewhat of a fœculent tinge: at 2, they became decidedly bilious, but containing many floculent shreds. At 10, urine was voided for the first time: at about which time it was noticed by an attendant, how wasted and shrivelled the fingers appeared, and on examination the toes appeared so likewise; the digits of both extremities feeling at the same time inflexible and contracted.—4, p.m. Since 2 o'clock, a little moisture has been felt about the feet and palms of the hands, the feeling, having since become proportionately comfortable, and spirits buoyant; there is still a little præcordial oppression experienced, but less thirst; a little appetite, too, is now felt; tongue slightly coated; the pulse, too, has much increased in volume, and diminished in frequency—in the recumbent posture it is 84, sitting up in bed increases it 20 beats in the minute, and giddiness is felt.—8, p.m. Half a cupful of broth was taken at 4 o'clock, but the feelings were, an hour after, that it had better been omitted; an occasional wine-glassful of water only, has therefore since been taken, and which it is the intention to continue throughout the night; the stools are frequent, watery and bilious; præcordial oppression greatly relieved, and feelings generally comfortable.

Repet. Pil.

18th, 6 a.m. The air being calm and oppressive, the attendance of a servant to fan the patient was required throughout the night—by which he rested, and slept for a few hours at intervals, having to get up frequently to stool: the evacuations continue as limpid as water, are green, but contain some mucus and a few floculent shreds; they are passed without griping, but occasion a good deal of irritation about the anus: feelings comfortable; but on rising feels giddy and weak; pulse soft and weakly; skin a little moist. Some sago was now taken; and a dose of Cheltenham salts two hours afterwards. From this time the convalescence was rapid, the diet being light, and proportioned to the weak state of the digestive organs." 41.

The narration of this case will probably induce many of our readers to think that it was not one of very great intensity—not more indeed than what might and does often occur in the hot seasons of this and other temperate climates, where people happen to eat two dinners in one day—take a warm-bath before the digestion of the first dinner is completed—and oppose themselves to the raw air of a damp night, after the second dinner. We confess that an attentive perusal of this case, would not lead us to any decided idea respecting the cause of the epidemic cholera of India—nor yet to its treatment. The author acknowledges that "the remedies prescribed were dictated more by his feelings than by any preconceived opinion :" and he became convinced that the "præcordial oppression was from the congestive state of the stomach and bowels," since the stimulants afforded no relief, whereas the evacuations invariably did so, more or less. These evacuations, therefore, he is convinced were the chief objects to be sought in the treatment of cholera.

"Had I, on the contrary, restrained this, by opium and stimulants, which has been too commonly the practice, inflammation would have become developed, with its attending symptoms, burning heat, and extreme irritability of the stomach ; restlessness, and so forth ; or spasms with their exhausting influence ; or the absence of these symptoms, and the non-development of excitement from cerebral congestion." 42.

Mr. S. thinks there can be no doubt that the disease was true cholera. The sero-mucous evacuations, the suddenness of the invasion, the prostration of strength, taken in conjunction with the state of the weather, leaves, he imagines, no question on this point. We certainly have our doubts respecting the degree of the disease, and consequently respecting the efficacy of the treatment—which was, indeed, little more than combating occasional symptoms or feelings as they occurred.

The fourth chapter is on MALARIA. By this term we mean a mephitic vapour, "the gazeous production of organized bodies in a state of decomposition."

"Dr. Dwight, an American divine and traveller, has made the nearest approach to the discovery of this subtile agent. When on his travels about the lakes of America, he found, that typhus did not prevail round the margin of lakes which were fed by natural springs, and which were bright upon their surface ; but on the contrary, it did prevail round the margin of those artificial lakes, which not being thus fed, were not only dull upon their surface, but covered occasionally by a dirty film, which, on experiment he found to be, the putrefactive product of animalculæ, which are existing in vegetable matter. For to this, as a variable agent, the conjoint operation of circumstances or system to its development—thus modifying the result, it is, that we must look to the effects, being in one case fever, and in another cholera.

A high or low temperature of atmosphere may be a modifying circumstance. And elevation of temperature may be a favourable predisposing cause to the result, being cholera, which has been so fatally prevalent of late years in India ; but it is not this simply, or the disease would be confined to such latitudes, which is not the case, as I shall adduce a most striking instance of its endemic prevalence, of recent occurrence in this country—at Clapham. It is true, this occurred in the month of August, but at a time when the thermometer was far from ranging high : and it may be further observed, that in places so situated, that is in high latitudes, fevers are of as common occurrence. The same objections obtain as to cold, or humidity, as well as to variety in the electrical condition of the atmosphere.

That malaria, I think it reasonable to suppose, is a variable compound, the consequences resulting—being thus modified. For were the nature of the disease to depend simply on quantity, or intensity of the same influence, we might expect the two diseases in an epidemic visitation of either, prevailing in common ; corresponding with the locality or particular circumstance of situation and the like, of the patient, which is not the case. Whereas, corresponding with intensity of influence, and susceptibility to impression, have we severity of grade of disease—whether of cholera, or of fever—marking their species or varieties. The connexion, however, between the two diseases is very intimate, for the fact is undoubted, that fever has not unfrequently succeeded, or has been conjointly prevalent with cholera during its epidemic visitations; though for a certainty it has not been of usual or common occurrence." 47.

There is another modifying circumstance which cannot escape observation —"cuticular capillary torpor, the effect of exposure, and which I believe to have been the immediate cause of my own attack." He does not, however, consider this state of the cutaneous circulation, whether resulting from simple exposure to cold, or to the influence of morbific exhalations, as essential to the development of cholera. He looks upon it rather as an accessory cause.

"For I believe the true state of the case will be found, that malaria giving rise to fever, in general is of a milder species; hence it is, that after its absorption, and the blood's contamination, its effects are not manifested in many cases till a distant period, or some accessory agency or exciting cause develops its action." 48.

In the primary actions of disease, the resemblance, says Mr. S., in all the essential characters between cholera and fever is very striking, and has been observed by various writers—the cholera appearing to many of them to be a protraction of the cold stage of fever. In proof that the disease, whether cholera or fever, may arise from animal exhalations alone, Mr. Searle appeals to a case published in the sixth Number of this Journal (quarterly series) where some men were instantly taken ill on opening a grave near Canton, the disease assuming a typhoid and even pestilential character. The observations of Fourcroy, and the experiments of Gaspard, Magendie, and others, are also adduced in support of this view. Still there is no doubt that it is to vegetable decomposition *chiefly* that we are to look for the agent which causes fevers, choleras, and very many other diseases.

In the second section of this chapter, Mr. Searle returns to his usual plan —that of extracting passages from the writings of others. Dr. Macculloch's work on malaria supplies our author with materials for the doctrine that cholera is produced from the invisible agent in question, and these materials are supported and augmented by various reports to the medical boards of the different presidencies of India. The author then copies from the Medical Gazette, the account of the fatal cholera at Clapham, caused apparently by the opening of a very foul drain in the back of the house where the disease occurred. These cases certainly support, in a very strong manner, the opinion, that cholera is the result of morbific exhalations from the earth.

"With the foregoing direct evidence I may venture to terminate the enquiry, and assume it as a fact, beyond controversy, that malaria is the cause of cholera, of the species we have now under consideration, which I beg to denominate congestive, as appearing to me the most appropriate with the general phenomena presented by the disease to our notice—in conformity with appearances on dissection—and as pointing out to us the most successful indications of practice to be pursued.

Since the above was put into the hands of the printer, a fact has been brought to my notice which I cannot resist mentioning; as it points out a source of the malaria kind, which would be little suspected. During the winter assizes at Hertford, four years ago, a boar-skin, which had a very offensive smell, having been introduced in evidence to the Court, many of the members, and other persons present on the occasion, were seized during the same night with cholera. The lady who related to me the circumstance, assured me, that three of the Council, who took up their abode in her house, were all of them attacked between the evening and the following morning, and were exceedingly ill. The medical gentleman who attended them, investigated the circumstances at the time, and declared this to have been the cause of their seizure; and which, we may add, is extremely probable." 69.

We now come to the fifth chapter. Mr. Searle, as an Indian practitioner, cannot be supposed as addicted to the art of book-making; yet, since the publication of Thornton's "PHILOSOPHY OF MEDICINE," we have never seen such pains taken to captivate the reader by a splendid display of "CONTENTS" at the head of each chapter or section; followed by such slender materials for satisfying the literary appetite so excited.

We regret to say that the two sections into which this fifth chapter is divided, have not produced that effect which they were evidently expected to do on the reader. "The chemical qualities of malaria—the effects of noxious gases on the system—the post-mortem appearances—the textures or parts on which malaria primarily operates—the source, nature, and distribution of the nervous energy—the action of the venom of the serpent—the primary operation of the febrile cause—its mode of action in producing synochal fever, &c." are little more than a tythe of the subjects discussed in eleven pages of large type—and that generally by extracts from various writers! To attempt to give any thing like a connected view of such heterogeneous materials, would defy much abler pens than we pretend to wield. We must, in this instance, imitate our *legerdemain* brethren (the *short-hand writers*, as defined by one of Mr. Wakley's witnesses) when they tell us that the speeches of certain members were "*quite inaudible*," but *understood* to be as follows:—the noxious influence of malaria is induced by the blood's contamination, which operates by "torpifying or arresting the chemical functions which take place in the general capillaries of the system, by which there is a diminished evolution of caloric and electricity, and, in consequence, debility of all the functions." This is supposed to take place in the same way as when the venom of the serpent and various other sedative poisons, animal and vegetable, are introduced into the system. For the arguments and illustrations in support of this position (the sum and substance of the fifth chapter) we must refer the curious inquirer to the work itself.

The four succeeding chapters, which are all theoretical, and very unsatisfactory, we shall pass over. The ninth chapter is thus headed. "Detailed treatment of Cholera in all its stages, and different varieties; written in a style adapted for non-professional readers, for the advantage of those more particularly, who are beyond the sphere of medical assistance."

It is certainly rather curious that the author should, all at once, descend from the most refined and erudite speculations on the nature and cause of cholera, to a non-professional code of instructions as to its treatment. By this transition, however, the author has gained one important advantage—that of becoming comprehensible. We shall allow him to speak for himself.

"The patient, on the first symptom of affection, to be confined to the recumbent posture, in short be laid in bed, between a pair of warm blankets, in an open airy room—when, should there be symptoms of indigestion, or the stomach appear oppressed, it will be advisable to evacuate it, by the patient either drinking plentifully of warm water, or by what is better—a mustard emetic, which operates as a powerful and general stimulant on the system; two table-spoonsful of the flour of mustard, or the same quantity of the powder, of the common black mustard seed, (which is to be procured in every bazaar in India, and which I found to be decidedly more active than the Europe flour) given in half a pint of pretty warm water, operates effectually in a few minutes,* without inducing any subsequent feelings of exhaustion; on the contrary, the eyes sparkle, and a genial glow of warmth succeeds throughout the system, with proportionate increased vigour of the circulation.

After the operation of this, rather preparative measure—to the stomach's being placed in the best condition, to be acted upon by our remedies more particularly curative,—and in furtherance of the same, a warm clyster should be administered, consisting of a dessert spoonful of table-salt dissolved in a pint of warm water, and a spoonful of common, or castor oil; and the same should be repeated every half hour, or oftener—for by thus keeping the bowels excited, tranquillity of the stomach is insured, and the consequent retention of our remedies—the chief of which is calomel; but as this requires some time before it can be received into the system, and effect its operation—as the absorbing power of the stomach, or susceptibility to its influence—in this disease, the distinguishing character of which, being the subduction of all the powers of life—is greatly diminished, it becomes necessary to give it in proportionately large doses, and at the same time, in aid of its operation, and in support of the living powers—to administer occasional cordials. A scruple of calomel in powder should therefore be placed upon the tongue, and the patient gargling his mouth with a little brandy and water, should swallow it; but it must be observed, the quantity of the latter should at no time exceed three table-spoonsful, which may be in the proportion of one of spirit to two of hot water,—as a bulk of any fluid, in a delicate irritable state of the stomach, is invariably productive of its rejection.

If the case is urgent, the same dose of calomel may be repeated every hour; otherwise, in two hours; or if the patient is much improved, in half the quantity; and thus prolonging the interval, or reducing the quantity—it must be continued, according to the state of the patient, till bilious stools and urine are produced;—the spirit and water, or mulled wine, either; or where the system is very low, thirty drops of (sal volatile) aromatic spirits of ammonia, or of hartshorn in half a wine-glassful of water, may be singly, or alternately administered, every quarter or half hour; with the precaution before given, to avoid oppressing the stomach by undue quantity.

In addition to these means, if the skin is cold, warm flannels should be constantly applied; or if the skin is damp and the patient suffers by cramps in his legs and arms, the parts may be well compressed, and rubbed with the flannels besprinkled with hot salt. We have yet omitted to mention a very important remedy, one capable of producing much good, or no less harm—this is blood-letting—which, if the patient is an European, or native of pretty robust habit, should be early resorted to—if the pulse admits of it, that is, if compared with another person's—it is of pretty moderate strength: the object to be borne in mind by bleeding in this case, is to excite, by removing oppression from the brain and circulation, and not to subdue the action of the heart, that it should be taken from the patient whilst continuing in the recumbent posture,—and here I must insist once for all, that on no account, and for no purpose, is the patient to be permitted to sit up, or leave the recumbent state, or sickness almost immediately takes place; the evacuations should therefore be received in a bed-pan, or cloth; and the blood be taken from a rather small orifice, that, the stream being in consequence small, the system may have time to accommodate itself to the deprivation,—the effect of which, however, should be carefully watched—the operator keeping his finger during the time on the pulse, at the

* This is an excellent dog medicine, a spoonful operates upon their bowels effectually in a few minutes.

same time encouraging the patient by suitable conversation;—when, at the instant it is found to flag, without reference to the quantity withdrawn, whether much or little, the finger should be placed over the orifice; but it must be borne in mind, that fear, nausea, or sickness may occasion this result, that should the quantity taken have been small, after a few minutes—if the pulse recovers its wonted strength, as it is an object to carry it to as great an extent as the circumstances of the patient admit—the finger may be removed from the orifice in the vein, and the blood allowed again to flow, with the precautions before specified; but should, after a further small loss, the same result ensue, it is clear that any additional attempt at this time would be injurious; though it may be afterwards practised, as excitement becomes developed, either in relief of spasms, sense of burning heat in the stomach, or pain in the head, or oppression of breathing; and with the precautions I have given, may be frequently put into practice, and without the possibility of harm—but, on the contrary, with the happiest effect; for in this disease small bleedings in relief of the engorgement of the brain, stomach, and heart, are clearly and most forcibly indicated. (See case A. in the Appendix.) The same intention is partially fulfilled by the clysters, but as warmth and excitement become developed, evinced too by the desire the patient has for cold water—these may be aided, or superseded by a weak and cold solution of Cheltenham or Epsom salts, or of cream of tartar, with which the patient may be now indulged—in the quantity of a wine-glassful at a time, instead of the cordials—which would now prove injurious; these will not, however, supersede the calomel, the necessity for which still continues, not only till bilious stools are procured, but even then, though in smaller doses, till healthy evacuations follow. It may however now, on febrile symptoms taking place, be well to combine it, with an equal weight of James's fever, or antimonial powder, and give it, if it is preferred, in the form of pill; but mind if the calomel is thus combined, acids, such as cream of tartar, are not admissible, as an emetic compound would be the result. The calomel and antimonial powder we would now advise, in the proportion of two grains of each, every two hours, with a tea-spoonful of Epsom, or Cheltenham salts, in a claret-glassful of water with every second dose: and if there is much thirst, the patient may, at the same time, be allowed a wine-glassful of barley or cold water every half hour; and the same be continued, till the secretions of bile and urine are restored—when, and not before, may the patient be allowed some sustenance, the best of which will be *light* beef tea, or chicken broth, for it must be remembered, and borne in mind, during the convalescence, that in proportion to the feeble state of the patient, so is the stomach weak and powers of digestion.

Many have an objection to salts; where this is the case, two table-spoonsful of castor oil may be substituted, or a dose of rhubarb and magnesia when this is preferred. Should the operation of the purgative be attended with much exhaustion, it may be necessary to support the patient with some spiced broth, wine and water, or mulled wine; or it may even be necessary to moderate it if there is much sinking—by a dose, of from twenty to forty drops of laudanum; but this is providing against contingencies, which with moderate care and attention will seldom be found necessary.

The secretions from the bowels are now sometimes so exceedingly acrimonious, that in passing along the line of bowels and from the anus, they produce, from extreme irritation—very considerable exhaustion; when this is the case, it will be advisable to inject an occasional emollient clyster, of starch or thick conjee water, with oil; to the first of which may be added a tea-spoonful of laudanum, and this repeated if necessary; at the same time hot flannels may be applied to the belly.

In the treatment recommended, we have had in view an ordinary attack of the disease, and coming on with symptoms of indigestion, or stomach derangement; should however the disease, which it not unfrequently does, have made an insidious approach, under mask of a simple loose state of the bowels, and from the continuance of which the patient is much exhausted; in a case of this sort it may be necessary to quiet the system, and arrest further action of the bowels, till a certain degree of excitement of the system becomes developed; this may be effected, by adding a grain of opium to the first dose of calomel, or an equal proportion (thirty drops) of laudanum, and which it may be necessary to repeat, but it is only under these and the like circumstances that opium can be recommended.—The treatment in other respects becomes the same, save that, in such cases, rhubarb and magnesia, or castor oil is to be preferred to salts, in the stage wherein these are recommended; and that bleeding and clysters can only become necessary, in the relief of the symptoms we have pointed out, when excitement becomes developed; excepting, when there is experienced a sense of oppression and distention about the stomach and bowels, when the clysters may be advisable; otherwise it is to be feared, the stomach will be affected with sickness, which will obviate all our attempts at relief. Should there be burning heat of stomach, while at the same time the body is death-like cold, it will be in vain to attempt resuscitation with stimulants, calomel is the only one admissible,—the stomach being in a state of inflammation, a scruple may be given every hour, and at the same time, as there is a great desire for cold water, and Nature's craving should be respected, a table-spoonful or two may be allowed every five or ten minutes, but not more at a time, as the stomach is in this state very irritable, and were a quart to be given, it would not satisfy the patient, who would desire as much more five minutes afterwards; that this precaution is indispensable. The clysters are also in this case to be continued; and should the sense of heat in the stomach be great, a dozen leeches may be applied over the part, and after their removal a flannel wrung out in warm water may be applied, and with this covering exposed to the air, so as to encourage a little evaporation if grateful to the feelings of the patient; it should however be observed, that the temperature be not too much reduced in this way. If the pulse admits of it, or as general excitement becomes developed, bleeding may be resorted to, particularly if there are spasms, with the precautions I have already enjoined in having recourse to this remedy.

There is another remedy, simple in the extreme, always available, and speaking both from personal experience and observation, I may add, of powerful operation, which is the fan or hand punkah—it not only renews the air, but condenses it, and thus aiding the respiratory function, assists in no inconsiderable degree in supporting the actions of life; that I cannot too earnestly recommend its uninterrupted use from the earliest period of attack; it is, at the same time, exceedingly grateful to the patient." 121.

The foregoing extract, and indeed the whole work will convince Mr. Searle's readers that he is not a very practised writer. The composition, in a literary point of view, is very inferior;* but this we should not, in the slightest degree, object to, if the originality of views or importance of practical precept had answered the expectations which are excited by the author, in the title page, preface, and various parts of the work. We cannot say, however, that a perusal of Mr. Searle's book has given us any additional insight into the *nature or cause of cholera.* His therapeutical indications we have laid before our readers, for, as they are founded on experience or observation, they form by far the most valuable part of the work.†

---

* Since the above was written we have learnt that the author's health was so bad at the time when the work was printing, that he was unable to superintend the correction of the press.

† Nearly half the work, or at least 100 pages, are dedicated to an appendix, consisting of cases and observations "extracted from the report of the Madras

Medical Board." We grieve that Mr. Searle had not some friend to advise him in this publication. It is astonishing that his own common sense did not tell him how unlikely it was that such a plan would succeed in these times.

At the end of this Appendix is another, containing an Essay on Vital Temperature and Nervous Energy; which was submitted to the Medical and Physical Society of Madras—a society which appears to have been dissolved soon after its formation, for want of support. We are sorry to find that the "nervous energy"—or at all events, the professional zeal of our Madras brethren is not equal to that of their more northern countrymen at Calcutta!

Of the original Essay we are unable to take any analytical notice. The following extract will afford a specimen of the author's electrical reasonings.

"The medulla oblongata (from which all the nerves of sense I believe arise) is the organ appropriated to receive the impressions of the senses, and which it communicates to the mind, by the blending of its nervous fibres with those of the cerebral organ, in the medullary portion of the latter. It is very clear that the senses are united in function, as they are only thus conjointly under the power of volition, as a proof, I cannot will to see, without both hearing and smelling, nor have I the power to arrest the function of either singly, but the whole I can simultaneously, by going to sleep; and thus it is that the muscular nerves of the organs of sense arise also from the medulla, as they are united in effect; and hence also it is, that the medulla oblongata is formed, by the union of the crura cerebri et cerebelli, or at least, that they enter very largely into its composition, thus blending the animal functions or voluntary, with the mental in this organ, as they are conjointly in operation.

The state of being awake, or alive to all the impressions of sense, I am of opinion is that, in which the current of electricity passes through or is extended to the medulla oblongata, and thus are the senses and all the functions in a state of excitement; whereas, in sleep, the stream of electricity takes a different channel, or perhaps wholly passes off by the 8th pair, which, it will be remembered, arise from the inferior part of the medulla; hence it is that digestion and the secretive processes are much more active during the state of sleep, and that dreams, we may infer, are occasioned by the excitement of the intellectual organ, by the 8th pair not withdrawing a quantity of electricity equal to the supply; meaning, of course, the quantity over and above what is required for the excitement of the respiratory muscles, which are at all times in operation; and, as their action is voluntary, it is very clear the organ of volition can never be at rest, like that of the mind and senses; and hence it is that we often turn over and move unconsciously during sleep; and this consideration affords some insight into the state of somnambulism. Finally, the spinal marrow appended to and surmounted by the cerebellum, are organs appropriated to volition, and the numerous functions more particularly characterizing animal existence.

In conclusion, it may be observed, that the scale of animal life comprises three conditions of existence. First—the organic, or that going on in the capillary system, or structure analogous; this, bordering on vegetable life; and the latter, on chemical affinity; rendering the last, the bond of union between organic and inanimate nature.

Second—the nervous and sentient, dependent on the first—or the more particularly animal, connected with organs of sense and volition.

The third and last surmounting the whole, the intellectual; on the summit of which, man stands proudly pre-eminent, with a brain or cerebral organ, developed in a ratio with his vast comprehension, and superiority of mind, above the rest of the Almighty's creation; and holding communion with an immortal spirit, in a way inconceivable to the limited number of faculties, with which, it has been the pleasure of the Almighty to endow him."

